# SSD with multi-scale feature fusion and attention mechanism

**DOI:** 10.1038/s41598-023-41373-1

**Published:** 2023-12-04

**Authors:** Qiang Liu, Lijun Dong, Zhigao Zeng, Wenqiu Zhu, Yanhui Zhu, Chen Meng

**Affiliations:** 1 College of Computer Science, Hunan University of Technology, Zhuzhou, Hunan China; 2 Intelligent Information Perception and Processing Technology Hunan Province Key Laboratory, Hunan University of Technology, Zhuzhou, Hunan China

**Keywords:** Engineering, Mathematics and computing

## Abstract

In the field of the Internet of Things, image acquisition equipment is the very important equipment, which will generate lots of invalid data during real-time monitoring. Analyzing the data collected directly from the terminal by edge calculation, we can remove invalid frames and improve the accuracy of system detection. SSD algorithm has a relatively light and fast detection speed. However, SSD algorithm do not take full advantage of both shallow and deep information of data. So a multiscale feature fusion attention mechanism structure based on SSD algorithm has been proposed in this paper, which combines multiscale feature fusion and attention mechanism. The adjacent feature layers for each detection layer are fused to improve the feature information expression ability. Then, the attention mechanism is added to increase the attention of the feature map channels. The results of the experiments show that the detection accuracy of the optimized model is improved, and the reliability of edge calculation has been improved.

## Introduction

The application scenarios of image acquisition equipment are more and more owing to the booming development of the Internet of Things and the huge breakthrough and progress of computer vision related technologies. On account of the increase of equipment nodes, the pressure of data transmission increases sharply. the data flow generated by image acquisition equipment may be very large, so it is necessary to use edge computing^1–3^ to preprocess images. With the successive breakthroughs of deep learning technologies^4–8^ and the rapid development of the world economy, object detection algorithms^9–11^ have made significant progresses in various fields^12–15^. Especially, target detection is one of the basic research projects in the fields of public transportation^16^, national defense and military. It is widely used in aerospace^17^, robot navigation^18^, industrial detection^19^, pedestrian tracking^20^ and military applications^21^. And a high-performance target detection algorithm can promote the development of industry. The so-called target detection is to find the target from pictures or videos by analyzing the geometric characteristics of the target, judge the specific category of each target accurately, and provide the bounding box of each target. The CNN (Convolution Neural Network)^22^ has been proved to be an effective model for processing visual tasks. The convolutional layer can capture the image representation of hierarchical patterns and obtain the feature layer of different receptive fields. To find a more powerful expression is an significant topic in the research of object detection, so that the network can better capture the significant information in the specific tasks^23–26^. The accuracy of edge equipment in screening images data is improved and the reliability is enhanced. In recent years, deep learning has developed rapidly. And more and more scholars have applied deep learning to the field of object detection. There are two kinds of target detection algorithms based on deep learning^27^. One is the object detection algorithm based on candidate box represented by RCNN^28^, Fast-RCNN^27^, Faster-RCNN^29,30^ etc. This kind of target detection algorithm firstly uses the Selective Search^31^, Edge Boxed^32^ and other algorithms to generate the candidate regions (region proposal)^33^ that may contain the target to be detected, and then these candidate regions are classified and located to achieve the intent of targets detection. The other one is the regression-based object detection algorithm represented by SSD^34–36^series and YOLO^37,38^ series. The regression-based object detection algorithm is surely faster than the box-based detection algorithm, and the main advantage of the box-based target detection algorithm is its high accuracy. Object detection algorithms not only require high precision, but also require fast real-time performance. Although the target detection algorithm based on candidate box has high precision, the generation of candidate box will consume a lot of time and result in unsatisfactory speed. However, the object detection based on regression does not need to generate candidate boxes and it is directly detected on the original image. The speed is greatly improved, but the disadvantage is that the accuracy is not high enough. With the improvement of the algorithm, some regression-based target detection algorithms have high accuracy and fast detection speed, and their accuracy is even higher than the same box-based target detection algorithms^39–41^. In this paper, the SSD target detection algorithm has been optimized, the extracted feature map has been divided into three categories according to the receptive field. Different scale fusion methods were carried out according to the categories to achieve a much better utilization of feature information and to enhance the robustness of the detection frame. Then, the channel attention mechanism is added to the fused feature layer to make the model become more interested in the specific channel information and to improve the network performance in channel dimension. The validity of proposed model is demonstrated by comparative experiment results, and the results show that the algorithm proposed in this paper not only improves the detection accuracy, but also ensures the detection speed^42–46^. In this paper, Senet (squeeze- and -exception networks)^47^ is used as the channel attention mechanism. The remaining structure of this paper is arranged as follows. In chapter two, we first review the SSD algorithm and some model evaluation criteria. In chapter three, we focus on the improved SSD model. After that, the result of experiment and the corresponding analysis are given in the fourth chapter. Finally, the fifth chapter summarizes the work.

## Background and related work

Now many scholars both domestically and internationally are interested in the field of object detection, especially small object detection. And they have done a lot of research works and achieved good research results. For examples, in order to raise the detection efficiency of small objects, an improved multiscale feature fusion method is proposed in reference^48^, namely, the atrous spatial pyramid pooling-balanced-feature pyramid network is proposed for object detection. In particular, the atrous convolution operators with different dilation rates are applied to fully utilize context information, where the skip connection is employed to achieve sufficient feature fusions. In reference^49^, the authors show how Deep Learning may be used to reliably extract higher-level features and then fuse multi-scale features to identify eddies, regardless of their structures and scales. And the experimental results show that high target detection accuracy can be get by their method.

Next, this chapter will describe the basic idea of the traditional SSD algorithm and analyse the advantages and shortcomings of SSD algorithm in detail. Then, some model evaluation criteria will be introduced.

### SSD network structure

**Figure 1:**
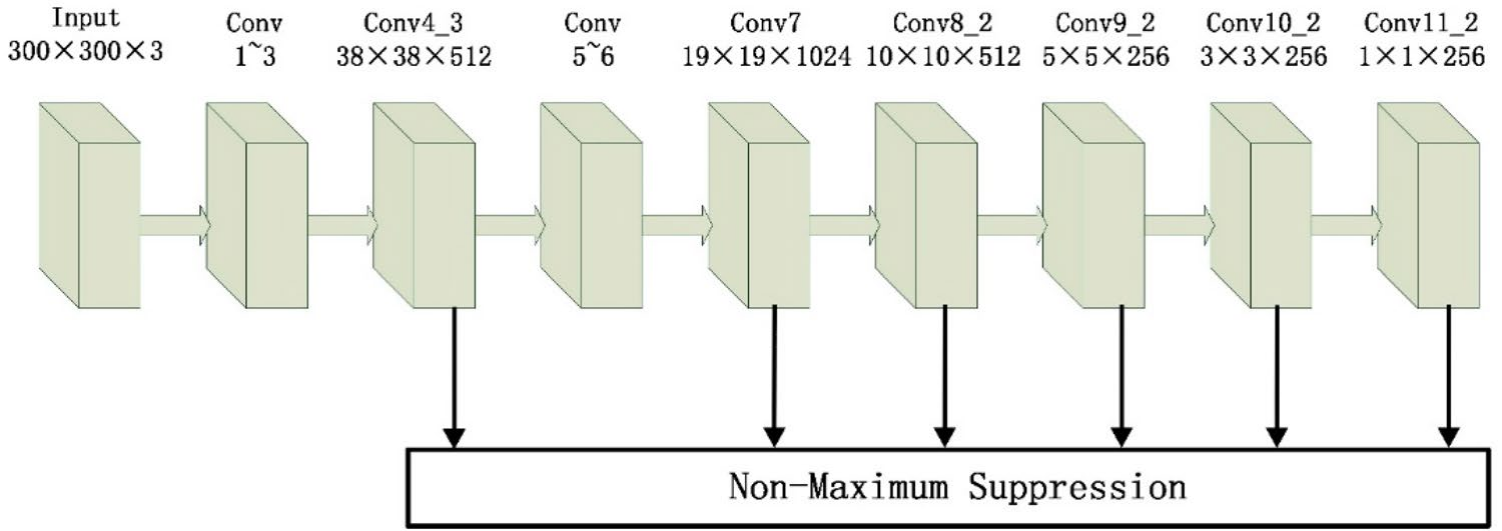
SSD structure chart.

SSD is one of the typical representatives of one-stage target detection algorithm. The model structure is shown in Fig. 1^50^. The image is adjusted by the algorithm to a fixed size 300 × 300, and they are inputted into the network. And use the backbone feature extraction network based on vgg16^51^ to obtain the feature layers of different scales, which are named Conv4_3 layer, Conv7 layer, Conv8_2 layer, Conv9_2 layer, Conv10_2 layer, Conv11_2 layer, and their sizes are (38 × 38 × 512), (19 × 19 × 1024), (10 × 10 × 512), (5 × 5 × 256), (3 × 3 × 256), (1 × 1 × 256). Then, each grid point of the feature layer creates some prior boxes with different aspect ratios. The numbers of prior boxes generated on different feature layers are different, which are 4, 6, 6, 6, 4, 4 respectively. On the basis of the size of the receptive fields, Conv4_3 layer and Conv7 layer have large size and small receptive fields and have strong geometrical information expression ability, which are used to detect smaller targets; Conv10_2 layer and Conv11_2 layer have small size and large receptive fields, and the semantic information expression ability is strong. It is suitable for the detection of large targets. The geometrical information and the semantic information obtained for Conv8_2 and Conv9_2 are between those obtained for Conv10_2 and Conv11_2, and they are used for detect medium targets. Finally, all redundant prior boxes are removed by non-maximum Supression (Non-Maximum Suppression, NMS) to generate the final detection boxes.

SSD algorithm uses high-level feature information with large receptive field to predict large objects, and low-level feature information with small receptive field to predict small objects effectively. This brings a problem: when the feature information of low-level network is used to predict small targets, SSD algorithm has a weak detection performance for small targets due to the lack of high-level semantic information because the deep feature map loss too much information and has insufficient resolution after being sampled multiple times.

### Loss function

Loss function of SSD algorithm contains two aspects: location loss ($L_{loc}$) and confidence loss ($L_{conf}$). There are many prior boxes, and relatively few objects to be detected from a image. Then many prior boxes cannot match a real box and cannot generate too many negative samples. This algorithm can conduct difficult sample mining, adjust and control the positive and negative samples, reduce the influence of too many negative samples, and improve the optimization speed and the stability of training results. The algorithm's loss function is defined as Eq. (1):

1 $$L\left( {x,c,l,g} \right) = \frac{1}{N}\left( {L_{cnf} \left( {x,c} \right) + \alpha L_{loc} \left( {x,l,g} \right)} \right)$$

The $L\left( {x,y,l,g} \right)$ is total loss function, $N$ is the number of default boxes which are matched truth boxes; the parameter $\alpha$ is used to adjusting the ratio between the location loss and the confidence loss; $c$ is category confidence;$l$ represents the positional information of predictive boxes; $g$ on behalf of the positional information of truth boxes; the value of input $x_{i,j}^{p} \in \left\{ {0,1} \right\}$ depends on the IoU (intersection over union, IoU)^52^ threshold between prior box and real box. When the IoU between a priori box *i* and real box *j* is greater than the threshold, $x = 1$. This indicates a priori box *i* is matched with real box *j*, and real box category is $p$, or $x = 0$. And the location loss function is adopted for Smooth L1 loss, the function is defined as Eq. (2):

2 $$L_{loc} \left( {x,l,g} \right) = \sum\limits_{i \in Pos}^{N} {\sum\limits_{m \in cx,cy,w,h} {x_{ij}^{k} } } smooth_{L1} \left( {l_{i}^{m} - \hat{g}_{j}^{m} } \right)$$

The $smooth_{L1}$ is defined as Eq. (3):

3 $$smooth_{L1} \left( x \right) = \left\{ {\begin{array}{*{20}c} {0.5x^{2} \quad if\left| x \right| < 1} \\ {\left| x \right| - 0.5\quad otherwise} \\ \end{array} } \right.$$

The $cx,cy$ are on behalf of the offset of boxes’ center along direction *x* and *y*; and the width and height of boxes represented by $w,h$; $i \in Pos$ shows the predictive box $i$ which is positive sample, and $Pos$ represent positive sample collection. Because the predictive box is encoded, so by encoding operation of the real box to get $\hat{g}$. The coding process defines as follows:

4 $$\hat{g}_{j}^{cx} = {{\left( {g_{j}^{cx} - d_{i}^{cx} } \right)} \mathord{\left/ {\vphantom {{\left( {g_{j}^{cx} - d_{i}^{cx} } \right)} {d_{i}^{w} }}} \right. \kern-0pt} {d_{i}^{w} }}$$

5 $$\hat{g}_{j}^{cy} = {{\left( {g_{j}^{cy} - d_{i}^{cy} } \right)} \mathord{\left/ {\vphantom {{\left( {g_{j}^{cy} - d_{i}^{cy} } \right)} {d_{i}^{h} }}} \right. \kern-0pt} {d_{i}^{h} }}$$

6 $$\hat{g}_{j}^{w} = \log \left( {\frac{{g_{j}^{w} }}{{d_{i}^{w} }}} \right)$$

7 $$\hat{g}_{j}^{h} = \log \left( {\frac{{g_{j}^{h} }}{{d_{i}^{h} }}} \right)$$

The confidence loss adopts softmax loss, which is defined as Eq. (8):

8 $$\begin{gathered} L_{conf} \left( {x,c} \right) = - \sum\limits_{i \in Pos}^{N} {x_{ij}^{p} \log \left( {\hat{c}_{i}^{p} } \right)} - \sum\limits_{i \in Neg} {\log \left( {\hat{c}_{i}^{0} } \right)} \, \hfill \\ where\;\hat{c}_{i}^{p} = \frac{{\exp \left( {c_{i}^{p} } \right)}}{{\sum\nolimits_{p} {c_{i}^{p} } }} \hfill \\ \end{gathered}$$

$i \in Neg$ represent the predictive box $i$ which is negative sample, and $Neg$ represent the negative sample collection. $\hat{c}_{i}^{0}$ is the probability which represents the category is correctly classified as background, the $\hat{c}_{i}^{p}$ calculated through the softmax function represents the probability that the category is correctly classified as non-background.

### Model evaluation criteria

Some model evaluation criteria are introduced. To evaluate the detection effect of the model, the following criteria are used to measure the model. And the common terms are shown in Table 1.

**Table 1:** Common terms for object detection evaluation criteria.

True positive (TP)	Number of positive samples which are classified correctly
True negative (TN)	Number of negative samples which are classified correctly
False positive (FP)	Number of positive samples which are classified correctly
False negative (FN)	Number of negative samples which are classified correctly

1. Accuracy: The accuracy is one of the common evaluation criteria of object detection model. The mathematical meaning is to divide the number of correctly classified samples by the number of all samples. The higher the accuracy, the better the detection effect of the model. And the function is as follows:

9 $$accuracy = \frac{TP + FN}{{TP + FN + FP + TN}}$$

2. Precision: The precision is calculated from the test results, which indicates the number of real positive samples in the samples predicted as positive samples. It is denoted as Eq. (10):

10 $$presision = \frac{TP}{{TP + FP}}$$

3. Recall: The recall rate is calculated from the real sample set, which indicates the probability of correct recognition in all positive samples. It is denoted as Eq. (11):

11 $$recall = \frac{TP}{{TP + FN}}$$

4. AP (average precision): In general, precision and recall rate are contradictory standards. Thus, AP is proposed to better measure the performance of the model. After drawing the smooth PR curve (precision recall curve), and the final AP value is calculated as follows:

12 $$AP = \int_{0}^{1} {P_{smooth} } \left( r \right)dr$$

5. mAP (mean average precision): AP means the average precision for a single category, while mAP means the average of AP for multiple categories. The value range of mAP is 0–1, and the higher the value of mAP, the better the detection performance. This criterion is the most important one in the evaluation criteria of object detection algorithm. It is denoted as follows:

13 $$mAP = \frac{AP}{{num\;classes}}$$

6. PFS (frames per second): Object detection algorithm requires high precision and fast detection speed. The ultimate goal is to find a high-precision and efficient model. The mathematical meaning of FPS refers to the quantity of pictures that the model can detect per second.

## Improved algorithm based on SSD

In this chapter, we will optimize the SSD algorithm and introduce the optimization steps in detail. There are two main steps to optimize the model. The first step is adopting different feature fusion methods for different scale feature layers to improve the utilization rate of feature maps. The second step is adopting the channel attention to optimize the model.

### Multi-scale feature fusion

**Figure 2:**
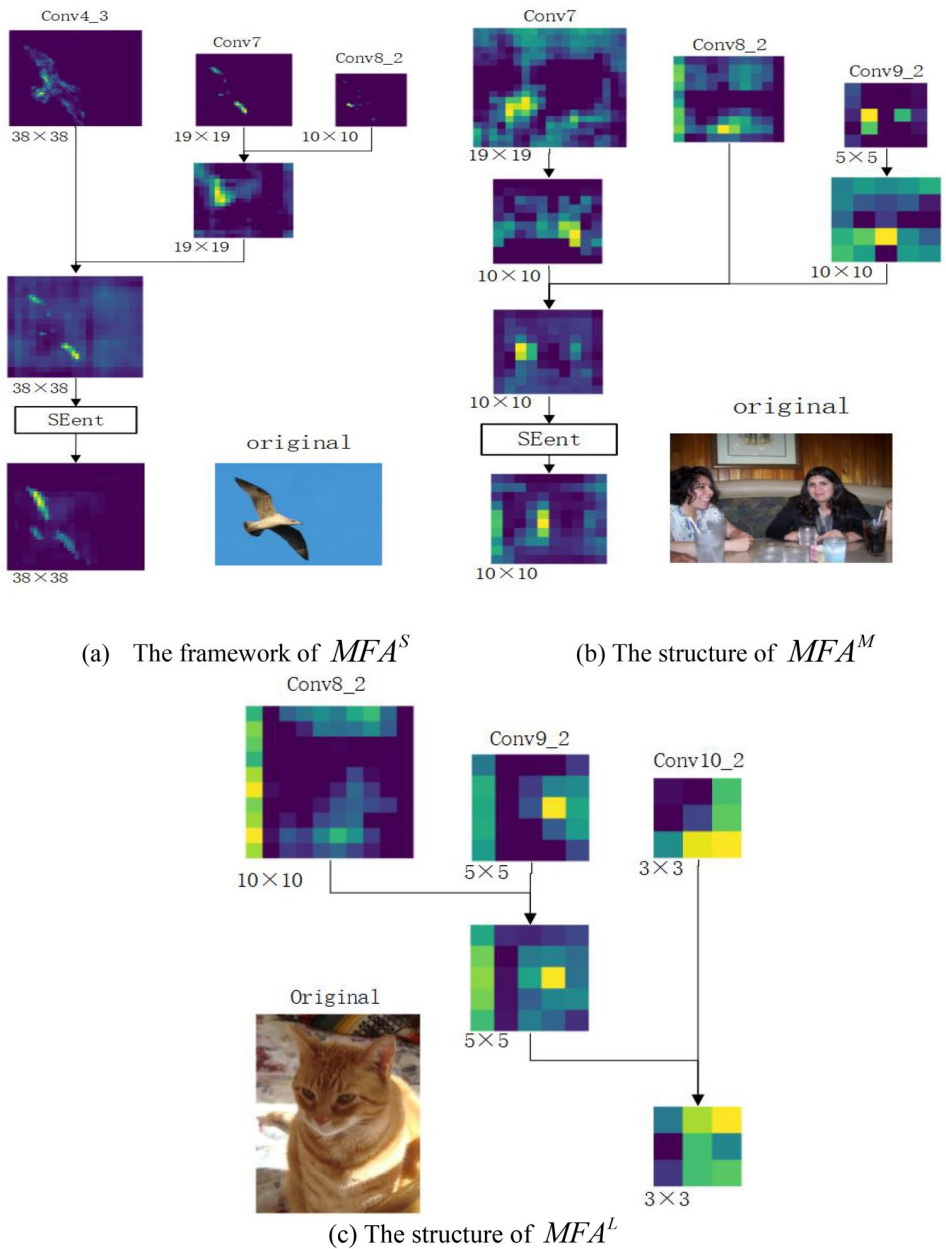
Visualization for $$MFA$$.

**Figure 3:**
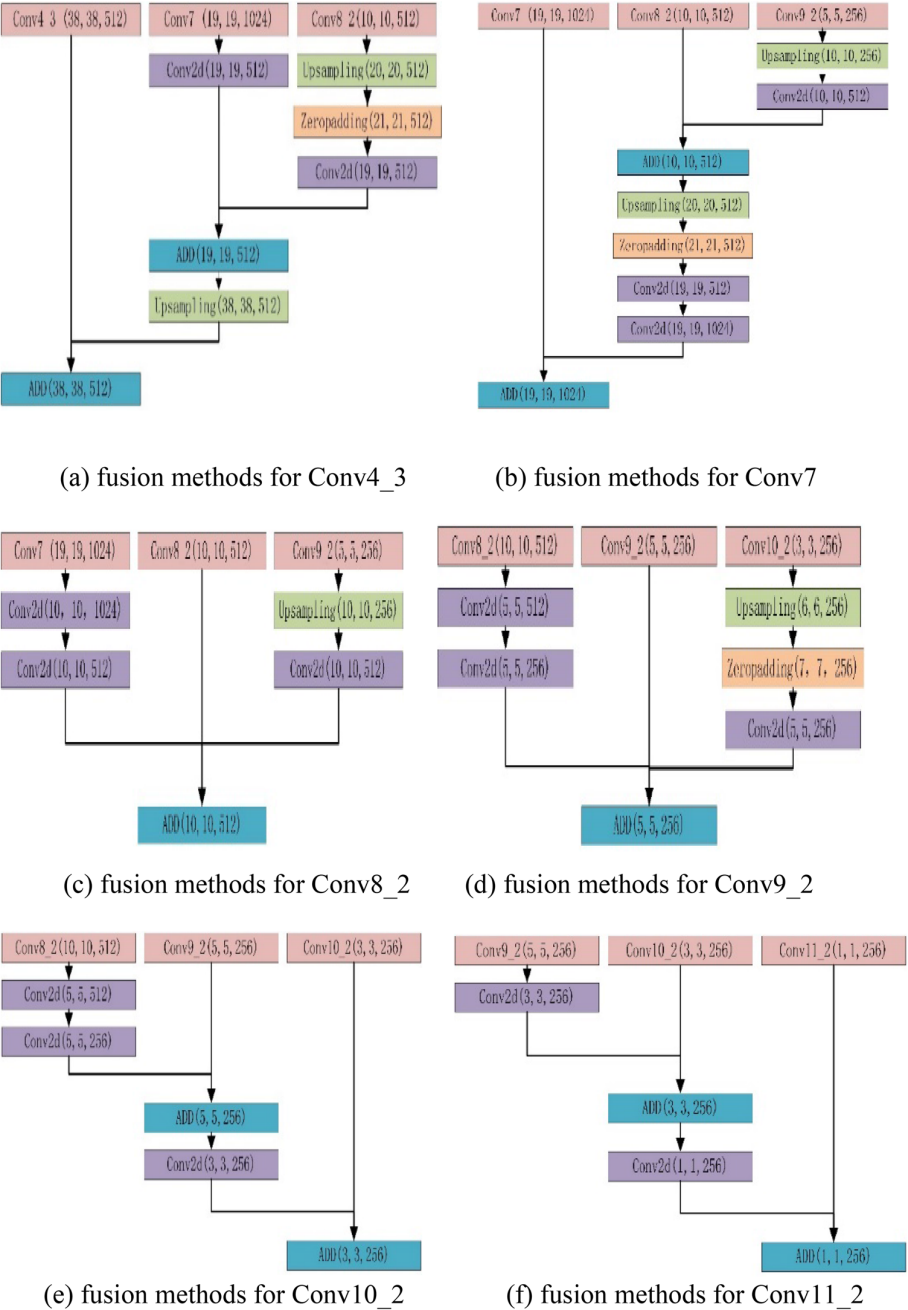
Fusion methods for different feature layers.

Based on the basic structure of SSD, multi-scale feature fusion attention mechanism ($MFA$) is proposed to improve the utilization rate of the model for extracting features. Different fusion mechanisms are adopted for feature layers of different sizes, the layer Conv4_3 for the detection of small targets is fused with Conv_7 and Conv8_2, and the fusion method can be seen in Fig. 3a. The fusion method of Conv7 which is fused with Conv8_2 and Conv9_2 is shown in Fig. 3b. It is beneficial to strengthen semantic information of the shallow feature layer by fusing the features of relatively deeper layer, and increased the accuracy of small target detection. There, we select any dimension of the corresponding feature layer for visualization, as shown in Fig. 2a. In the thesis, we named method multi-scale feature fusion attention ($MFA^{S}$) for Small object. While the Conv8_2 used to detect medium targets is fused with Conv7 and Conv9_2, the fusion method can be shown as Fig. 3c. And the fusion method of Conv9_2 which fused with Conv8_2 and Conv10_2 can be seen in Fig. 3d, making full use of the information from adjacent extracted features to improve the ability of information expression. Feature fusion operation to detected medium-sized targets is called Multi-scale feature fusion attention ($MFA^{M}$) for medium object, the visualization results are shown in Fig. 2b. Finally, layer Conv10_2 used for detecting large-scale objects is fused with Conv8_2 and Conv9_2, and the fusion method can be seen in Fig. 3e. And the fusion method of layer Conv11_2 fused with Conv9_2 and Conv10_2 can be seen in Fig. 3f. As the deep feature layer goes through multiple convolution and downsampling, the receptive field becomes larger, but lots of feature information are lost, which affects the detection accuracy, especially for smaller objects. Such influence can be reduced through the fusion of relatively shallow features, and such operation is named multi-scale feature fusion attention ($MFA^{L}$) for large object. Specific fusion steps are visualized as shown in Fig. 2c. In the fusion step, we change the size of feature maps by upsampling and convolution with a stride size of 2, and adopt the convolution with kernel 1 × 1 to change the number of channels. The persons and animals in Fig. 2 are from reference^53^.

In this paper, different fusion methods are adopted for different depth feature layers, which greatly improves the utilization rate of feature information. To reduce the overfitting of the model, the following random data enhancement was performed on the original data to improve the diversity of the input data. (1) zoom: randomly scale the image to a certain size; (2) flip: randomly flip the picture from side to side; (3) color replacer: transform the image from RGB color space to HSV color space, and fine-tune the image's hue (H), saturation (S), and value (V). test results of different fusion methods on the PASCAL VOC 2007 datasets are shown in Table 2 and $MFA$ is $\sum\nolimits_{i \in S,M,L} {MFA^{i} }$.

**Table 2:** Performance comparison between different methods.

	SSD	SSD + $MFA^{S}$	SSD + $MFA^{M}$	SSD + $MFA^{L}$	SSD + *MFA*
$mAP$ (%)	87.30	90.05	89.78	89.56	90.57
FPS	29.31	26.75	27.62	29.06	26.11

The Table 2 indicates that FPS of the real-time detection of different fusion methods is lower than that of the conventional SSD algorithm to a lesser extent. And $\mathrm{mAP}$ mAP of the SSD algorithm of different fusion methods are higher than the conventional algorithm of SSD, and $\mathrm{mAP}$ of SSD algorithm with $MFA$ is 90.57%, increasing 3.27% compared with the conventional SSD algorithm. The average detection speed of SSD algorithm with $MFA$ on the experiment platform is 26.11 frame/second, compared to the conventional SSD algorithm reduced 3.2 frame/second.

### Feature channel attention mechanism

**Figure 4:**
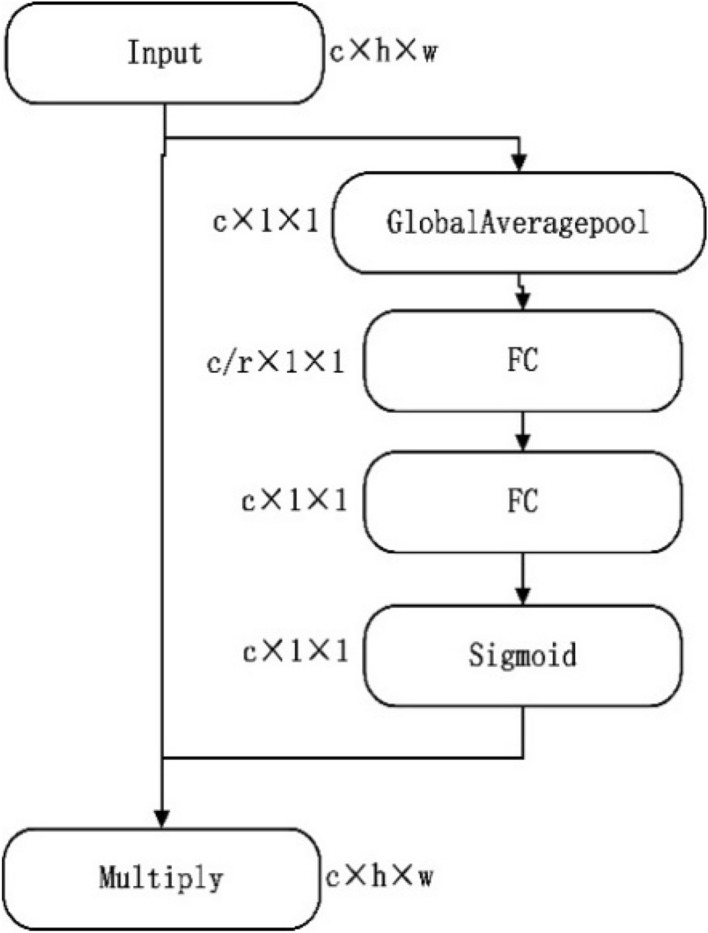
Senet structure chart.

Squeeze-and-excitation network(SEnet) were proposed by Senior R&D engineer Hu Jie and his team, the network won the Image Classification task champion of the last ImageNet 2017 with great advantage. SEnet network structure is shown in Fig. 4.

**Figure 5:**
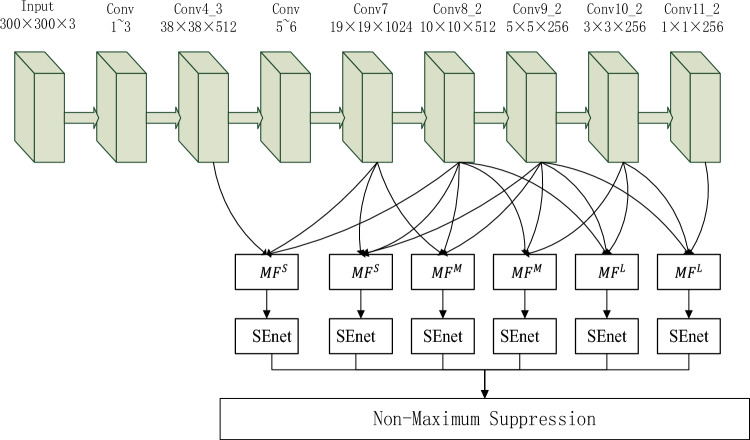
Improved SSD structure diagram.

SEnet alters the attention between feature channels to improve model feature extraction. By learning to automatically acquire the importance of each characteristic channel, according to this degree, more attention is paid to the model's effective channel, while the ineffective or inefficient channel is suppressed. SEnet consists of two important parts, squeeze and excitation. The operation of squeeze is to compress each two-dimensional data into a real number through global average pooling in spatial dimension, and the real number has a global receptive field. Next, we learn to generate weights for each channel, which names excitation. SEnet network parameters increase mainly comes from two full connection layers, and the first full connection layer through the compression ratio *r* (*r* = 16) reduced the number of arguments. Therefore, the detection rate of the proposed algorithm is only slightly reduced. In this study, SEnet was added to each feature layer after different fusion operations, and the framework of the peoposed SSD algorithm was shown in Fig. 5.

## Experimental results and analysis

### Experimental equipment and data

The experimental equipment configuration in this paper is as follows:

Intel(R) Core (TM) i5-9300HF CPU @ 2.40 GHz, 16 G memory;

GPU: NVIDIA GeForce GTX 1660 Ti, 16 G memory;

Operating System: Windows10;

Program running environment: python 3.7.7, tensorflow2.2.0, CUDA10.1;

Dataset: PASCAL VOC 2007 (http://host.robots.ox.ac.uk/pascal/VOC/voc2007/)^53^, and the experiment data table can be seen in Table 3.

**Table 3:** Experiment data table of PASCAL VOC 2007.

	Train set		Test set	
	Images	Objects	Images	Objects
Aeroplane	402	547	40	44
Bicycle	434	613	48	77
Bird	536	833	76	112
Boat	313	492	40	61
Bottle	412	888	44	86
Bus	327	400	33	42
Car	1314	2244	120	207
Cat	588	652	71	78
Chair	774	1412	88	142
Cow	240	450	28	53
Diningtable	360	391	30	30
Dog	729	874	110	125
Horse	498	619	63	91
Motorbike	425	614	42	50
Person	3632	8300	383	918
Pottedplant	427	908	42	86
sheep	175	453	18	46
Sofa	397	431	55	56
Train	462	514	58	65
Tvmonitor	443	581	42	51

### Analysis of experimental results

In this paper, four common target detection algorithms, namely, SSD, YOLOv3, YOLOv4, and Faster RCnn, are used to compare the performance with the improved SSD algorithm. And Table 4 shows the experimental results.

**Table 4:** The performance comparison of the different object detection algorithms.

	${\mathrm{mAP}}_{50}$ (%)	${\mathrm{mAP}}_{50:90}$ (%)	mAP75 (%)	*FPS/s*
SSD	87.30	49.17	35.19	29.31
YOLOv3	79.69	47.89	44.63	17.76
YOLOv4	88.57	51.81	49.99	15.45
Faster-RCNN	79.28	53.06	54.18	1.56
SSD + *MFA*	90.57	64.96	69.00	26.11

Experiments are carried out using PASCAL VOC 2007 dataset and detection performance indexes include mAP and FPS. $\mathrm{mAP}$ is the average of all kinds of classes’$\mathrm{AP}$, FPS is detection speed. ${\mathrm{mAP}}_{50}$ refers to the average precision when IoU threshold of the real box and prior box is 0.5. And ${\mathrm{mAP}}_{75}$ refers to the average precision when IoU threshold of real box and prior box is 0.75.The ${\mathrm{mAP}}_{50:90}$ is the average of ${\mathrm{mAP}}_{50}$, ${\mathrm{mAP}}_{60}$, ${\mathrm{mAP}}_{70}$, ${\mathrm{mAP}}_{80}$, ${\mathrm{mAP}}_{90}$. The experimental data show that the $\mathrm{mAP}$ mAP of improved algorithm of SSD (SSD + MFA) under different IoU threshold is the highest. With ${\mathrm{mAP}}_{50}$ as evaluation standard, the improved SSD algorithm is 2.00% better than the second-ranked YOLOv4 algorithm.With ${\mathrm{mAP}}_{75}$ as evaluation standard, the improved SSD algorithm is 14.82% higher than the second-ranked Faster RCNN algorithm. With ${\mathrm{mAP}}_{50:90}$ as evaluation standard, the improved SSD algorithm is 11.90% higher than the second-ranked Faster RCNN algorithm. The improved SSD algorithm is second-ranked in average detection speed, and it’s average detection speed is only 3.2 frames /second lower than the SSD algorithm ranked first, however, the average detection of the improved SSD algorithm is 8.35 frames/second higher than the third-ranked YOLOv3 algorithm. The comprehensive comparison shows that the improved SSD algorithm has the best performance.

**Figure 6:**
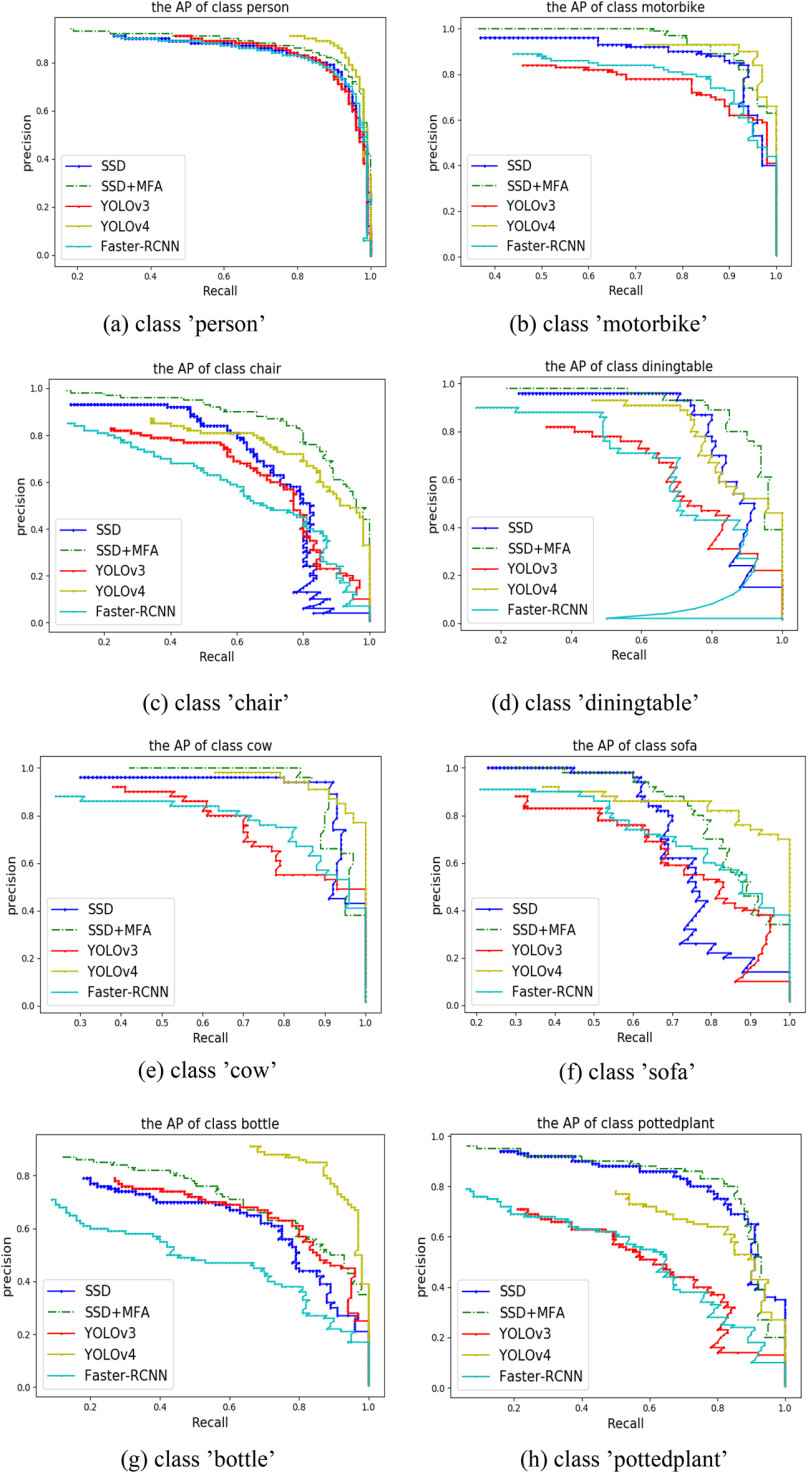
Comparison of five algorithms in different categories of accuracy—recall rate curve.

Figure 6 shows the accuracy rate, namely, recall rate curve comparison diagram of the average precision of different algorithms in different categories. Where SSD stands for conventional SSD algorithm, SSD+$\mathrm{MFA}$ MFA stands for improved SSD algorithm, YOLOv3 stands for YOLOv3 algorithm, YOLOv4 stands for YOLOv4 algorithm, and Faster-RCNN stands for Faster-RCNN algorithm. Seen from the figure, for the class' person' and class 'motorbike', there is a small difference in the accuracy rate—recall rate curve of each detection algorithm, but the improved SSD algorithm has the best performance. And the accuracy rate—recall rate curve of the improved SSD algorithm in class 'chair' and class 'dining table' increases significantly, which’s AP respectively are 88%, 92%, the detection accuracy is clearly better than other detection algorithms. For class 'cow' and class 'sofa', the improved SSD algorithm, SSD algorithm, and YOLOv4 algorithm have a small difference in detection accuracy, but are significantly better than YOLOv3 algorithm and Faster RCNN algorithm. For the class 'bottle', YOLOv4 algorithm has the highest precision, and improved SSD algorithms’$\mathrm{AP}$ ranks second. For the class 'pottedplant', the detection accuracy of the improved SSD algorithm and YOLOv4 algorithm is clearly better than other algorithms. To sum up, the detection effects of the improved SSD algorithm in different size targets both have been improved.

**Figure 7:**
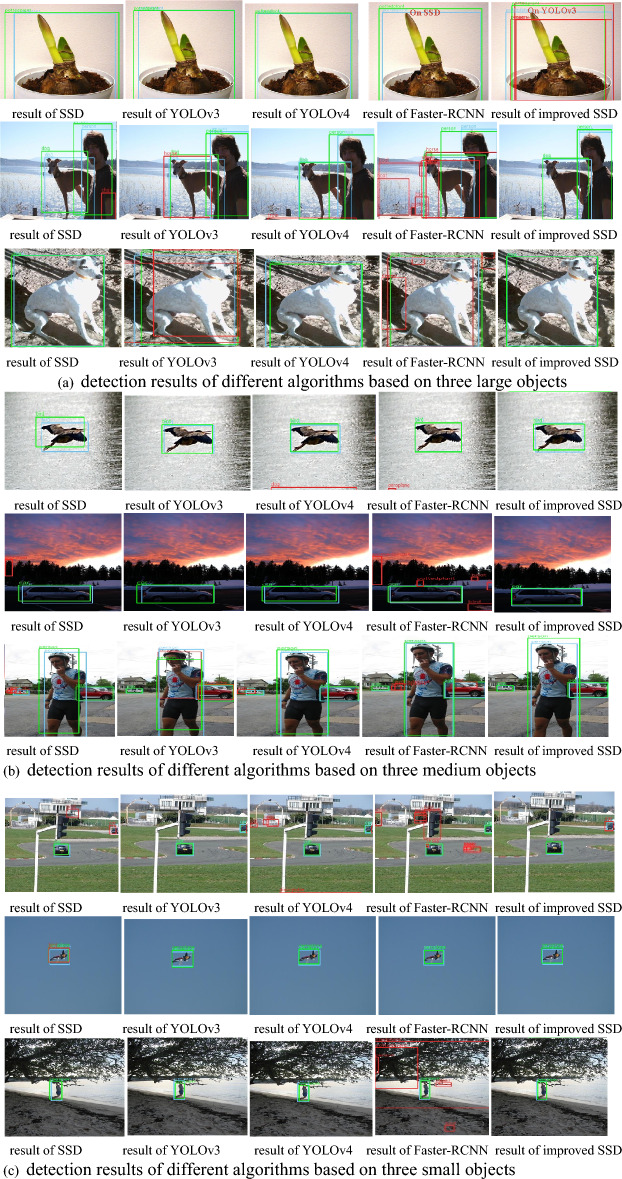
Detection results of different algorithms on PASCAL VOC dataset.

Compared with the other four algorithms, the improved SSD algorithm can reduce the error detection box and improve the detection accuracy. The comparison of detection results between different detection algorithms and the improved SSD algorithm are shown in Fig. 7. Select three detection results of large objects, three detection results of medium objects, and three detection results of small objects, respectively, among which the red boundary boxes are the error detection box, the blue boundary boxes are the real box, and the green boundary boxes are the prediction box corresponding to the real box. The results show that the mAP of the improved SSD algorithm are improved for objects of different scales. In addition, the error detection box is significantly reduced, and the IoU between the detection result (prediction box) of the improved SSD algorithm and the corresponding real box are also improved. The persons and other objects in Fig. 7 are from reference^53^.

## Conclusion

In this paper, an improved SSD algorithm (SSD + MFA) is proposed by adopting different fusion methods for feature extraction different scales layers and using the channel attention mechanism to reallocate the channel weights of the fused feature map. The $\mathrm{mAP}$ on PASCAL VOC2007 dataset reached 90.57%, which is 3.27% higher than the conventional SSD algorithm and 2.00% higher than YOLOv4 algorithm. The improved SSD algorithm can effectively reduce the error detection rate.

And the value of mAP of detection targets for different sizes has been improved to some extent, which improved significantly the precision of edge equipment screening image.

### Ethics approval and consent to participate

The research is approved by the College of Computer Science, and Intelligent Information Perception and Processing Technology Hunan Province Key Laboratory, Hunan University of Technology, Zhuzhou, Hunan, China, 412007. The research includes some information and images. All subjects involved in the information and images, or their guardians agreed to for publication of identifying information/images in an online open-access publication.

## Data Availability

The datasets used or analysed during the current study are available from the corresponding author on reasonable request.
